# Reliability of humeral head measurements performed using two- and three-dimensional computed tomography in patients with shoulder instability

**DOI:** 10.1007/s00264-020-04710-x

**Published:** 2020-07-26

**Authors:** Jakub Stefaniak, A. M. Kubicka, A. Wawrzyniak, L. Romanowski, P. Lubiatowski

**Affiliations:** 1 Sport Trauma and Biomechanics Unit, Department of Traumatology, Orthopaedics and Hand Surgery, Poznań University of Medical Sciences, Poznań, Poland; 2 Rehasport Clinic, Poznań, Poland; 3 Institute of Zoology, Poznań University of Life Sciences, Poznań, Poland

**Keywords:** Shoulder instability, Measurement reliability, Bone defects, 2D-CT, 3D-CT

## Abstract

**Purpose:**

The aim of the study was to compare two measurement methods of humeral head defects in patients with shoulder instability. Intra- and inter-observer reliability of humeral head parameters were performed with the use of 2D and 3D computed tomography.

**Methods:**

The study group was composed of one hundred humeral heads measured with the use of preoperative 2D and 3D computed tomography by three independent observers (two experienced and one inexperienced). All observers repeated measurements after 1 week. The intra-class correlation coefficient (ICC) and the minimal detectable change with 95% confidence (MDC_95_%) were used for statistical analysis of diagnostic agreement.

**Results:**

For 3D inter-observer reliability, ICC values were “excellent” for all parameters and MDC_95_% values were “excellent” or “reasonable.” All intra-observer ICC and MDC_95_% values for 3D were “excellent” for experienced and inexperienced observers. For 2D-CT, ICC values were usually “good” or “moderate” with MDC_95_% values higher than 10 or 30%.

**Conclusions:**

Three-dimensional CT measurements are more reliable than 2D for humeral head and Hill-Sachs lesion assessment. This study showed that 2D measurements, even performed by experienced observers (orthopaedic surgeons), are burdened with errors. The 3D reconstruction decreased the risk of error by eliminating inaccuracy in setting the plane of the measurements.

## Introduction

Shoulder instability is a common condition affecting 25 in every 100.000 people a year. It affects mainly young people, especially men (3:1) [1]. In people under 20 years of age, the risk of recurrent instability after the first dislocation can be up to 90% [2].

During anterior shoulder dislocation, the humeral head is displaced in front of the glenoid and its posterior surface is wedged into the anterior edge of the glenoid. The resulting bone impression, called Hill-Sachs defect, is the common diagnosis in patients with recurrent shoulder instability. The presence of a Hill-Sachs defect may predispose to a conflict between the humeral head and glenoid, and consequently to the dislocation of the shoulder joint [3].

Diagnosis of the defects of the anterior glenoid rim and the Hill-Sachs defect is important in treatment and facilitates the selection of an appropriate treatment method [4, 5]. Recently, significant attention has been paid to the issue of coexistence of humeral bone defects with glenoid defects. Not only the presence of a humeral head defect but also its morphology, location, and its interplay with glenoid bone loss might matter from the biomechanical point of view [6, 7].

Currently, the golden standard in the treatment of patients with shoulder instability is arthroscopic Bankart repair, which in patients with normal morphology of the glenoid and humeral head proves to be highly effective and with a low risk of recurrence of instability [4]. However, in the case of glenohumeral bone defects, the effectiveness of the soft tissue repair method drops significantly. Both glenoid and humeral head defects have been considered as the most important risk factors that should guide us in our selection of the most appropriate technique to stabilize the shoulder in case of instability [8–10].

Hence, correct tools for bone evaluation are needed. Instability severity index score (ISIS) developed by Balg and Boileau uses a quite simple way of X-ray evaluation [8] for definition of the defects. However, Bouliane at al. found there to be very low inter-rater accuracy in the approach [11, 12]. According to Hirshmann et al. [13], conventional X-ray is characterized by poor reliability, two-dimensional computed tomography (2D-CT) by medium reliability, and three-dimensional computed tomography (3D-CT) by high reliability of assessment.

We have been able to show in our previous study that 3D glenoid reconstruction is more reliable for glenoid bone loss assessment than 2D [14]. Therefore, we have focused on the measurement of humeral head defects in shoulder instability.

### The aim

The aim of this study was to assess the reliability of the most common methods to measure humeral head and its defects in patients with recurrent shoulder instability using 2D-CT and 3D-CT. We have hypothesized that 3D-CT will be more reliable than 2D-CT in assessing humeral head deficiency. In order to test the hypothesis, we have performed intra-observer and inter-observer tests by experienced and inexperienced evaluators. This study is a continuation of previous approach to evaluate the glenoid defects [14].

## Material and methods

One hundred consecutive CT scans performed on 100 patients (mean age 35.5; SD 15.5; min 17; max 69) diagnosed with traumatic anterior shoulder instability were obtained from our radiology department. The scans were collected by independent orthopaedic surgeons who did not participate in the assessment of method reliability. All the patients underwent a physical examination in order to identify other shoulder pathology like rotator cuff tears, deformations, fractures, or osteoarthritis. In the next stage, a basic radiology assessment showed that 63 of the 100 CT scans displayed signs of shoulder instability (63 glenoid bone loss, of which 47 Hill-Sachs defects).

Each of the CT scans was subjected to appropriate computer processing depending on the type of measurement method used: 2D-CT with multiplanar reconstruction and 3D-CT reconstruction. For both techniques, several of the most frequently measured indices of humeral head and Hill-Sachs lesion were chosen for further assessment (Table 1): circle area of humeral head, Hill-Sachs length, Hill-Sachs depth, humeral head length, humeral head height, anatomical neck width.

**Table 1:** Measurements of humeral head

Name of measurement	Description	References
Circle area of humeral head	A circle is fitted with its dimensions to the edge of the joint surface	Saito et al. [15] Cho et al. [16]
Hill-Sachs length	The length of Hill-Sachs erosion	Kodali et al. [17]
Hill-Sachs depth	The length measured between the deepest point of Hill-Sachs and the border of circle area of humeral head measurement	Kodali et al.
Humeral head length	Measurement performed from the articular surface of the humeral head to the greater tubercle, perpendicular to the long axis of the humerus	Pearl et al.
Humeral head height	Measurement performed from the articular surface of the humeral head perpendicular to the line of the anatomical neck of the humeral head	Pearl et al. [18, 19]
Anatomical neck width	Measurement performed along the line of the anatomical neck of the humeral head on the sagittal plane	Pearl et al.

All related measurements were performed by three independent observers. Two observers were orthopaedic surgeons specializing in shoulder surgery. The third was an observer with no experience in medical orthopaedic treatment (not a physician). Each of them performed measurements twice with a seven day interval without prior knowledge of the results of the first measurement and the findings of the second investigator.

### 2D measurement method

**Fig. 1:**
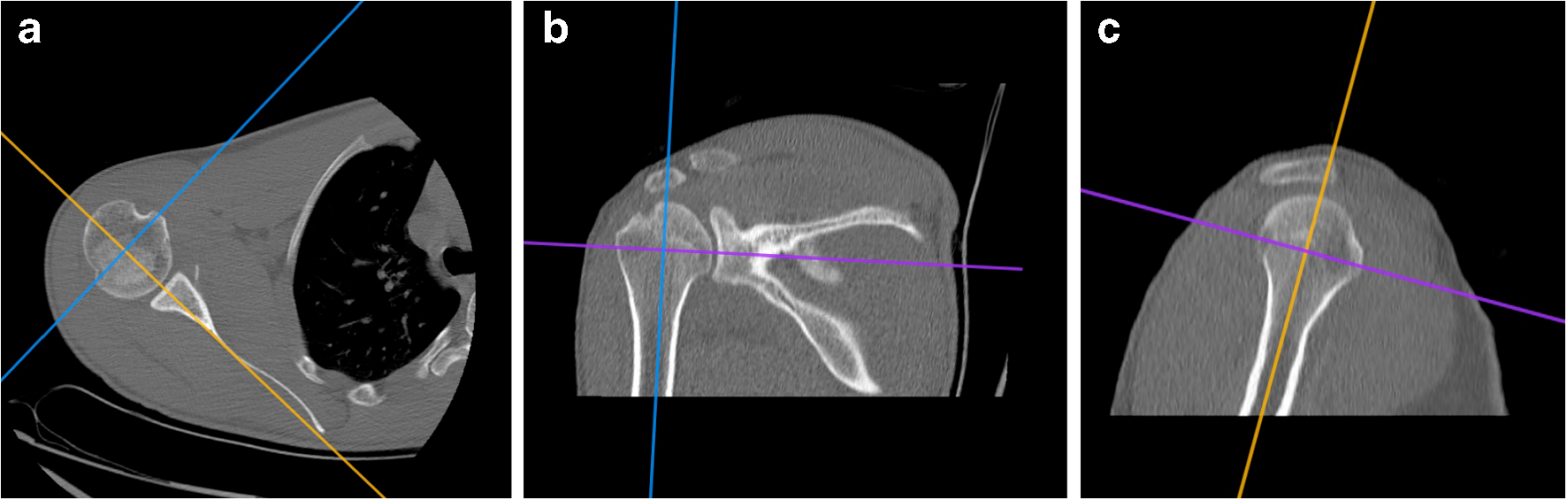
Initial 2D-CT multiplanar reconstruction for humeral head measurements. The sagittal (**c**) and frontal plane (**b**) axes run along the long axis of the humerus; the third axis on the transverse plane (**a**) marks the long axis of the humeral head (Osirix MD)

**Fig. 2:**
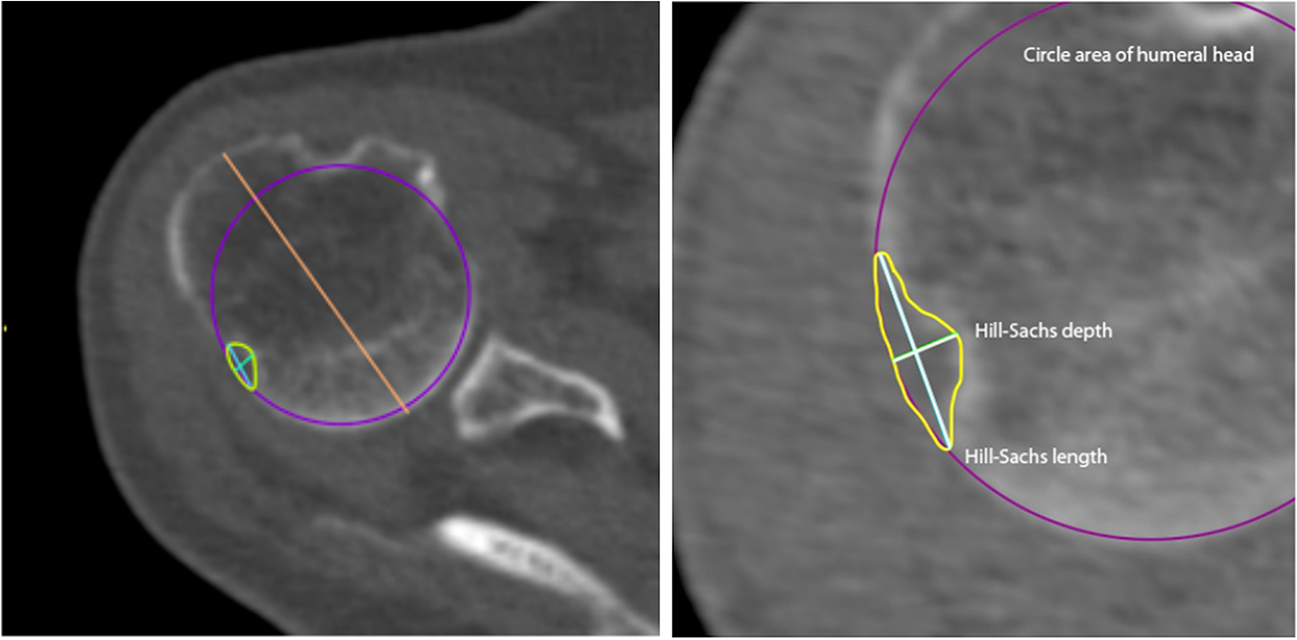
Measurements of humeral head on the transverse plane: circle area of humeral head, Hill-Sachs length, Hill-Sachs depth

**Fig. 3:**
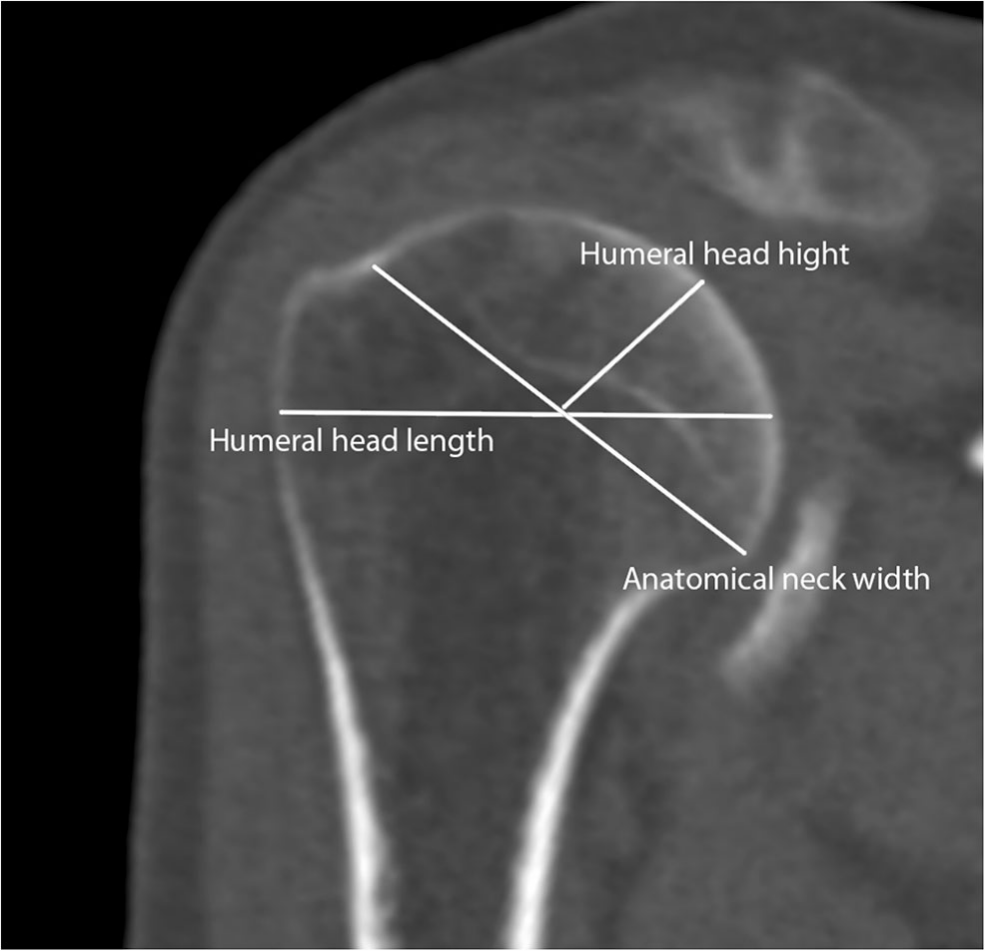
Measurements of humeral head on the sagittal plane: humeral head length, anatomical neck width, humeral head height (Osirix MD)

2D-CT method was based on an analysis of two-dimensional computed tomography with multiplanar reconstruction, using the OsiriX MD 64-bit software (v.8.5). In the first stage of the assessment, the shoulder CT scans were reconstructed in the 3D Curved-MPR module. Then, this image was set in three planes: frontal, sagittal, and transverse (Fig. 1) and measurements were performed (Table 1, Figs. 2 and 3).

### 3D measurement method

**Fig. 4:**
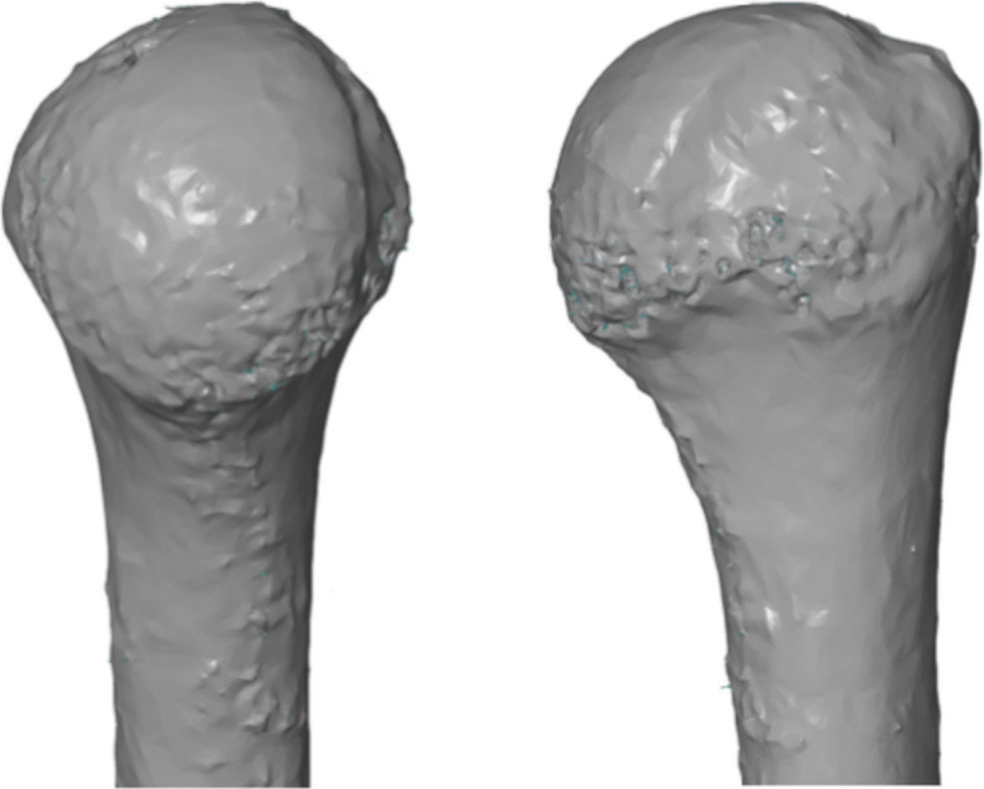
3D-CT reconstruction of humeral head in GOM Inspect (V8) software

**Fig. 5:**
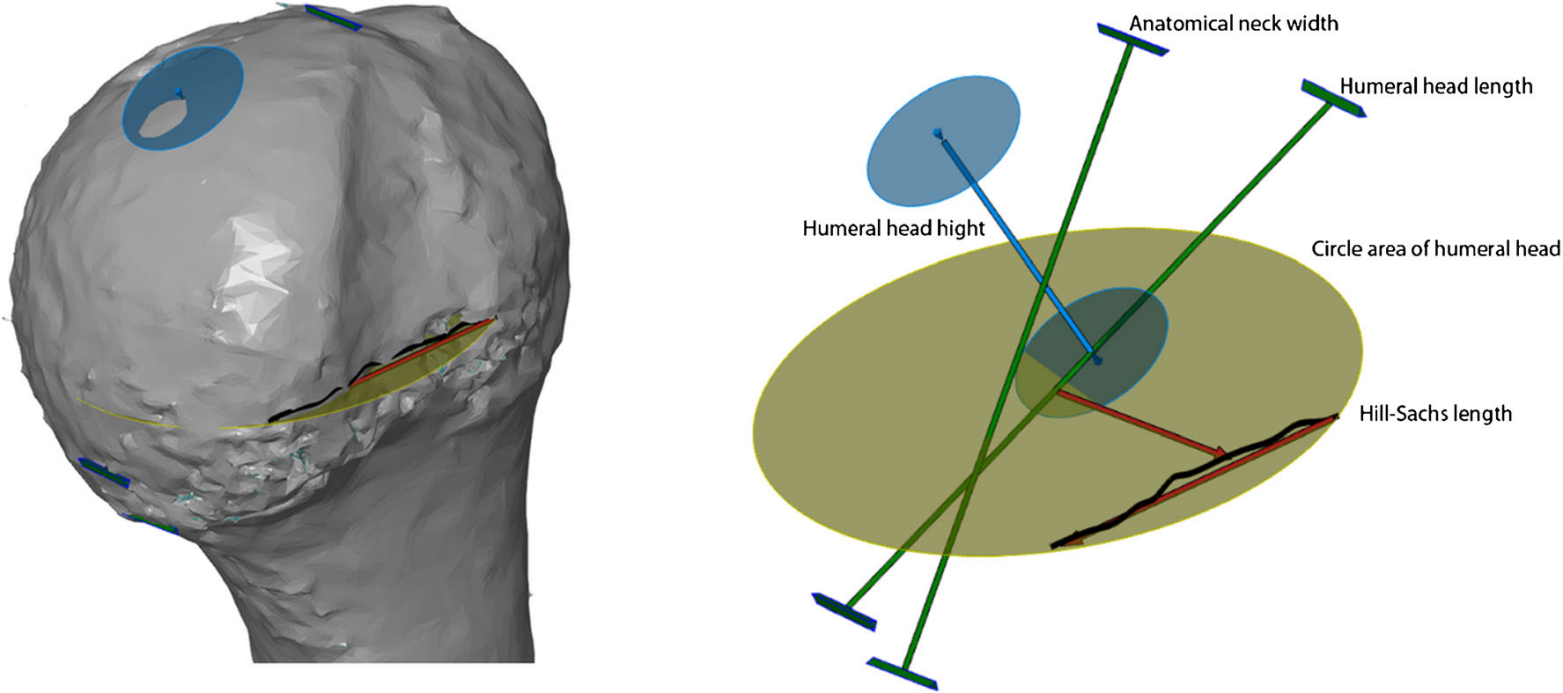
Measurements of humeral head: humeral head length, anatomical neck width, humeral head height, circle area of humeral head, Hill-Sachs length (GOM Inspect (V8) software)

3D-CT was based on CT analysis in three-dimensional reconstruction. In the first stage, all scans were reconstructed in three-dimensional space using the 3D Slicer software (3D Slicer ver 4.4). The program allowed conversion of a DICOM file into a Mesh file, which could then be further evaluated using the GOM Inspect (GOM, ver 8) software (Figs. 4 and 5).

3D reconstruction was conducted by one observer to avoid any errors associated with the 3D reconstruction itself. Thirty randomly chosen tomograms were reconstructed with an interval of seven days. One of the CT scans in pair was marked as “model” and converted into the CAD format, while the second tomogram, called “comparative,” was converted into the Mesh format. Both CT scans as a pair were compared with each other using the GOM Inspect (V8) program. The reliability of the 3D reconstruction was positively assessed, as the average differences within pairs did not exceed 0.15 mm.

Next, all measurements were performed on 3D-CT with the use of GOM Inspect program (Table 1).

### Statement of human and animal rights

This article does not contain any studies involving human participants and animals performed by any of the authors.

### Statistical analysis

In our study, we relied on two models of reliability testing: intra- and inter-observer reliability in which we choose three independent researchers with different levels of experience. In the process of developing of our study, we relied on existing research that concerned reliability assessment in measurement methods. Moreover, our study is the continuation of previous study, regarding the assessment of reliability of glenoid bone defects measurements on 2D and 3D CT [14]. However, in this study, we decided to add one more observer (orthopaedic surgeon) to present reliability of measurements performed by observers with the same level of expertise.

Statistical analysis was performed using Microsoft Office Excel (Microsoft ver. 16.23) and SPSS software (IBM ver. 22.0.0.1) and supported by professional statistician.

Calculation of sample size for reliability tests with intra-class correlation coefficient (ICC) was performed to check whether number of patients included in the analysis allows reliable statistical analysis [20]. In the intra-observer reliability, with the use of ICC, the sample size should not contain less than *N* = 13 and *N*_drop_ = 15 in case of 10% dropout (expected ICC 0.92; two repetitions; the lower acceptable ICC was 0.7 and a significance level for a one-tailed test was a = 0.05). For the inter-observer reliability, the sample size should not contain less than *N* = 38 with *N*_drop_ = 43 in case of 10% dropout (expected reliability ICC = 0.92; precision 0.05 with confidence level 95%).

Intra-observer and inter-observer reliability were calculated for 100 CT scans for humeral head measurements (circle area of humeral head, humeral head length, humeral head height, anatomical neck width) and for 47 CT scans containing visible Hill-Sachs lesion (Hill-Sachs length, Hill-Sachs depth). All measurements were repeated after seven days by each observer.

Reliability was calculated by means of ICC in which values can range from 0 to 1, where “0” means total non-compliance, and “1” absolute compliance of the measurement.

For inter- and intra-observer reliability, ICC (2,*k*) (two-way random effects, absolute agreement, multiple raters/measurements) model was calculated with a 95% confidence interval (95% CI) [21]. Compliance in the ICC range is ranked as follows: “excellent,” > 0.9; “good,” 0.75 < ICC <0.9; “moderate,” 0.5 < ICC < 0.7; and “poor,” ICC < 0.5 [21, 22].

The minimal detectable change (MDC) defined as the minimal amount of change that is required to distinguish a true performance change from a change due to variability in performance or measurement error. MDC with 95% confidence (MDC_95_%) was calculated as percentages of measurement mean and showed real change and repeatability of the test. MDC_95_% values lower than 30% were assessed as “reasonable” and lower than 10% as “excellent” [23].

## Results

### Inter-observer reliability

In 2D CT method, ICC values were “excellent” for parameters circle area of humeral head and Hill-Sachs depth; “good” for Hill-Sachs length, humeral head length, and humeral head height; and “moderate” for anatomical neck width. In 3D CT method, ICC values for all parameters were “excellent.”

For 3D measurements, the MDC_95_% values were “excellent” for circle area of humeral head, Hill-Sachs length, Hill-Sachs-depth, humeral head length, and humeral head height (2.76–2.89) and “reasonable” for anatomical neck width (14.00).

For 2D measurements, MDC_95_%values were excellent for circle area of humeral head and humeral head height (5.29–9.76) and “reasonable” for Hill-Sachs depth, humeral head length, and anatomical neck width (23.94–24.73). For Hill-Sachs length measurement, MDC_95_% value was higher than 30% (74.99).

All measurements are presented in Table 2.

**Table 2:** ICC values for inter-observer 2D-CT and 3D-CT measurements and statistical significance. ICC, inter-class correlation coefficient; 95% CI, 95% confidence interval

	*N*	2D			3D		
		ICC	95%CI	MDC%	ICC	95%CI	MDC%
Circle area of humeral head	100	0.965	0.983–0.983	5,29	0,988	0.912–0.995	2.76
Hill-Sachs length	47	0.825	0.595–0.991	74,99	0,984	0.949–0.993	2.83
Hill-Sachs depth	47	0.968	0.949–0.980	23,94	0,990	0.967–0.995	2.81
Humeral head length	100	0.786	0.701–0.846	12,69	0,986	0.963–0.993	2.83
Humeral head height	100	0.888	0.844–0.920	9,76	0,999	0.998–0.999	2.89
Anatomical neck width	100	0.735	0.511–0.841	24,73	0,991	0.985–0.994	14.00

### Intra-observer reliability

In 2D-CT method, ICC values for 1st experienced observer were “excellent” for Hill-Sachs length and Hill-Sachs depth, “good” for circle area of humeral head and humeral head height, and “moderate” for humeral head length and anatomical neck width.

All ICC values for the first experienced observer in 3D method were “excellent.”

MDC_95_% values were excellent for all 3D measurements (2.78–9.46) and reasonable for all 2D parameters (11.16–22.47).

For the second experienced observer in 2D-CT evaluation, ICC values were “excellent” for anatomical neck width, Hill-Sachs depth, “good” for circle area of humeral head and humeral head height, and “moderate” for Hill-Sachs length and humeral head length.

All ICC values for the second experienced observer in 3D CT method were “excellent.”

MDC_95_% values were excellent for all 3D measurements (2.79–8.66). For 2D measurements, MDC_95_% values were “excellent” for anatomical neck width and “reasonable” for circle area of humeral head, Hill-Sachs depth, humeral head height, and humeral head length (8.37–16.77). For Hill-Sachs length, MDC_95_% value was higher than 30% (44.34%).

For the inexperienced observer, in 2D CT method, ICC values were “good” for circle area of humeral head, Hill-Sachs depth, and humeral head height and “moderate” for Hill-Sachs length, humeral head length, and anatomical neck width.

All ICC values for the in-experienced observer in 3D-CT method were “excellent.”

All values of MDC_95_% were “excellent” for 3D measurements (3.44–9.18). For 2D measurements, MDC_95_% values were “reasonable” for circle area of humeral head, humeral head height, humeral head length, and anatomical neck width (11.87–25.21). No 2D parameters were “excellent.” Two parameters, Hill-Sachs length and Hill-Sachs depth, had MDC_95_% values higher than 30–44.35 and 63.37, respectively.

All results are presented in Table 3.

**Table 3:** ICC values for 1st and 2nd experienced and in-experienced intra-observer measurements. ICC, interclass correlation coefficient; 95% CI, 95% confidence interval

	*N*	Experienced observer 1						Experienced observer 2						In-experienced observer					
		2D			3D			2D			3D			2D			3D		
		ICC	95%CI	MDC%	ICC	95%CI	MDC%	ICC	95%CI	MDC%	ICC	95%CI	MDC%	ICC	95%CI	MDC%	ICC	95%CI	MDC%
Circle area of humeral head	100	0.867	0.815–0.905	11.16	0.987	0.982–0.991	2.79	0.877	0.828–0.911	11.17	0.987	0.978–0.992	2.79	0.876	0.827–0.911	11.87	0.981	0.973–0.986	3.44
Hill-Sachs length	47	0.978	0.964–0.986	11.60	0.999	0.998–0.999	2.89	0.720	0.545–0.828	44.34	0.999	0.866–0,996	2.90	0.724	0.552–0.831	44.35	0.997	0.995–0.998	4.41
Hill-Sachs depth	47	0.992	0.987–0.995	12.56	0.993	0.989–0.996	9.46	0.992	0.987–0.995	12.59	0.996	0.992–0.998	8.66	0.771	0.628–0.859	63.37	0.994	0.989–0.996	9.18
Humeral head length	100	0.595	0.417–0.715	22.47	0.985	0.979–0.989	2.78	0.750	0.651–0.820	16.77	0.979	0.968–0.986	3.21	0.741	0.639–0.814	18.19	0.975	0.965–0.982	3.45
Humeral head height	100	0.894	0.852–0.924	11.18	0.990	0.986–0.993	2.79	0.811	0.736–0.864	16.77	0.988	0.981–0.992	3.08	0.867	0.814–0.904	14.00	0.976	0.967–0.983	4.34
Anatomical neck width	100	0.592	0.422–0.710	25.17	0.986	0.981–0.990	2.78	0.925	0.896–0.946	8.37	0.981	0.971–0.987	3.21	0.641	0.489–0.746	25.21	0.978	0.969–0.984	3.47

## Discussion

Based on the results of the study, we have confirmed the hypothesis that computed tomography with 3D reconstruction is more reliable than 2D-CT for evaluation of humeral head parameters and bone defects. Furthermore, we have also shown that a 3D-CT evaluation seems to be resistant to bias resulting from the level of the researcher’s experience. In all evaluations, ICC values were “excellent” for all 3D-CT measurements. MDC_95_% values for 3D measurements were “excellent” for almost all parameters (except inter-observer anatomical neck width measurement, where the MDC_95_% value was “reasonable” (14.00)). For comparison, 2D measurements had usually good or moderate ICC values and “reasonable” or above 30% threshold values of MDC_95_%.

Bone defects on the lateral surface of the humeral head were first described in 1855 by Malgaigna [24], but only in 1940 did Hill and Sachs completely described and published the morphology of these defects [25]. The incidence of the Hill-Sachs defect increases with the number of shoulder joint dislocations. After the first episode of dislocation, Hill-Sachs presence is found in about 65% of cases, and in patients with recurrent instability in almost 93% of cases [26, 27]. In our series, the incidence of humeral head lesions was 47%. The exact assessment depends on the use of an appropriate diagnostic method. The presence of Hill-Sachs bone loss is important in the case of a risk of a conflict between the humeral bone defect and the anterior glenoid rim. Therefore, an accurate assessment of the morphology of the defect (length, width, depth, and location), which is essential due to its impact on the choice of treatment method, depends on the quality of the examination methodology [28].

The assessment of the bone defects of the anterior glenoid rim and humeral head is usually based on a two-dimensional analysis of transverse CT scans. Currently available software for computed tomography analysis provides a number of useful tools, with the help of which we can perform the necessary measurements such as a measurement of the length of a straight line joining two points, the surface area of the selected point, or volume of space. However, the analysis of a two-dimensional image of an essentially three-dimensional object leads to the risk of making a mistake resulting from image imperfections or measurement errors on the part of the person performing the measurement. Some models of two-dimensional image processing, available in commercial programs, gave the opportunity to reconstruct a stack of individual images into one three-dimensional model, the projection of which can be set in three planes (transverse, frontal, and sagittal). However, the analysis of such projections (application of measurements) is still carried out only on one plane, which will not eliminate the basic errors of the method [29, 30]. Referring to this type of “hybrid” image projection as a three-dimensional reconstruction is therefore not fully correct.

The real three-dimensional method assumes the reconstruction of a virtual three-dimensional model of the tested object and gives the possibility to perform such measurements in three planes. When applying measurements within the glenoid or the humeral head, the model can be freely rotated to identify and apply the correct measurement point. This reduces the risk of error arising from faulty setting of the initial measurement projection, as in the two-dimensional method [14].

In our previous study, we analyzed the reliability of the 2D and 3D measurement method of anterior glenoid bone loss assessment in patients with anterior shoulder instability [14]. We have proved that ICC values for 3D-CT reconstruction were significantly more reliable for most measurements than the 2D method. Just as in this study, we have proven that the 3D method allows for more accurate measurement by researchers with different levels of experience. Similar to the measurements of the glenoid defect, different measuring methods of the Hill-Sachs bone loss were described in the literature [31]. Kodali et al. positively assessed the reliability of the Hill-Sachs measurement by two-dimensional tomography, measuring the width and depth of the defect in three planes (sagittal, frontal, and transverse) [17]. In contrast, the method of three-dimensional tomography was used by Cho et al. assessing the width and depth of Hill-Sachs defects and their position relative to the articular surface of the humeral head [16]. Ho et al. assessed the reliability of 3D-CT measurements of nine anatomically shaped bone models of Hill-Sachs lesions. There was strong agreement between all raters for all measured parameters (length, width, depth) [32].

One of the most important findings in our study was the experience of evaluation in interpretation of CT images matters if is based on 2D images. The spatial view and 3D reconstruction seem to provide more relatable tools independent of the experience of the surgeon. This aspect of measurement methods has not been, to our best knowledge, studied in shoulder imagine evaluations up until now (the exception being our previous publication on glenoid defects [14]). Kaup et al. evaluated the impact of radiologists’ experience in diagnostic accuracy of osteoporotic vertebral compression fractures in CT and MRI imaging [33]. In another field of imaging, radiologists’ experience was also addressed in the assessment of salivary gland tumours with the use of CT and MRI [34]. In both studies, higher experience resulted in greater reliability.

Traditional X-rays have also been used for the evaluation of humeral head defects. They have been part of the commonly used ISIS. This score assists surgeons in identifying the risk factors for recurrence of shoulder instability following shoulder stabilization treatment. In the case of an absence of risks, arthroscopic Bankart repair has a high potential for effective treatment. Bone defects are the major criteria and misinterpretation may lead to underscoring and hence incorrect surgical planning. Burkhart et al. shows that in 67% of patients with an inverted-pear glenoid have recurrent shoulder instability after soft tissue repair and a 100% recurrence in patients with Hill-Sachs [4]. Tauber et al. found bone defects in 57% out of 41 patients re-operated on for recurrence of instability [9]. Finally, Boileau et al. identified risk factors for recurrence instability—attritional glenoid defect (> 25% bone loss) and Hill-Sachs with stretched anterior capsule or laxity [35]. The inexperience of the surgeon and the case of unclear image together with low value of instruments could be some of the reasons for such weak assessments. Traditional X-ray allows us to diagnose the presence of a defect only in about 7% of cases after the first dislocation episode, in comparison, computed tomography or magnetic resonance tomography images are much more accurate and allow us to determine the presence of a defect in more than 90% of cases [36]. Chalmers et al. report that linear measurements resulted in most aggressive recommendations of treatment [37]. Stillwater et al. assessed that there are no significant differences between measurements performed on 3D-CT and 3D-MR postprocessed images [38]. On the other hand, there are some studies which undermine the accuracy of 3D-CT measurements in comparison to measurements performed with the use of arthroscopy [39].

One of the limitations of the study is that we have just focused on humeral head defects. Recently, as studied by Di Giacomo et al. [40] and Yamamoto et al. [7], the importance of HSL the position (not only the size) and bipolar lesions have been found to play an important role in so called engagement. The identification of both seems to be an important factor in deciding on the choice of optimal operating technique to stabilize the shoulder. This study is a continuation of our work on glenoid evaluation. An evaluation of the interplay of bipolar lesions would exceed the scope of one research paper and is proposed as a matter for a further study.

Another weakness identified in current diagnosis methods is the complexity of 3D reconstruction measurements. 3D methods of measurement with the currently available software are relatively advanced and difficult to use accurately. As a result, it may be troublesome and time consuming in everyday clinical practice. An automated process could improve the practical use applicability of CT-based image reconstruction. Such attempts have already been implanted in surgical planning for arthroplasty. Good examples of this are patients-specific instruments (PSI) software used in hip, knee, or shoulder replacement (OrthoView software etc.).

To conclude, 3D-CT measurements are more reliable than 2D for humeral head and Hill-Sachs lesion assessment. This study showed that 2D measurements, even performed by experienced observers (orthopedic surgeons) are burdened with errors. The 3D reconstruction decreased the risk of error due to inaccuracy in setting the plane of the measurements and might be precise and easy to use for evaluators inexperienced in computed tomography assessment.
